# Large Language Models for Endodontic Symptom Assessment and Treatment Planning Using Image-Free Clinical Records: Comparative Evaluation Study

**DOI:** 10.2196/86145

**Published:** 2026-07-24

**Authors:** Dahyun Seo, Jieun Cheong, Yiseul Choi, Yooseok Shin, Wonse Park

**Affiliations:** 1 Department of Advanced General Dentistry, College of Dentistry, Yonsei University, Seoul, Republic of Korea; 2 Yonsei University, Institute for Innovation in Digital Healthcare, Seoul, Republic of Korea; 3 Department of Conservative Dentistry, Yonsei University College of Dentistry, 50-1 Yonsei-ro, Seodaemun-gu, Seoul, 03722, Republic of Korea, +82-2-2228-3146, +82-2-313-7575

**Keywords:** natural language processing, decision support systems, clinical, endodontics, prompt design, ChatGPT, Gemini, Microsoft Bing

## Abstract

**Background:**

Accurate assessment of pulpal status is essential for achieving successful endodontic outcomes. However, direct evaluation remains inherently challenging because the pulp is surrounded by calcified tissue, necessitating reliance on clinical and radiographic examinations for diagnostic and prognostic decision-making. These procedures demand substantial clinical expertise and time, and less-experienced clinicians often face challenges that may lead to errors in diagnosis and treatment planning. Recent advancements in large language models (LLMs) offer promising opportunities to enhance clinical reasoning by facilitating the integration of evidence and supporting methodical diagnostic decision-making.

**Objective:**

This study aimed to evaluate the clinical applicability of LLMs by comparing their text-based clinical screening performance and the clinical validity of their treatment plan responses with those of human evaluators.

**Methods:**

Between January 2011 and December 2022, 100 clinical cases involving primary endodontic disease were randomly selected from the clinical records of outpatients who visited the Department of Conservative Dentistry or Advanced General Dentistry (AGD) at Yonsei University Dental Hospital. Four prompt types, combining 2 variables (language and role), were used as input for 4 LLMs. Both LLMs and human evaluators (AGD specialists, AGD residents, endodontic residents, and senior dental students) assessed the cases using text-based clinical records. Radiographic images were not directly provided. Screening performance was evaluated using a 0-to-2-point concordance scale, and treatment plan validity and relevance were assessed using a 5-point Likert scale.

**Results:**

Among the 4 LLMs evaluated, ChatGPT achieved the highest mean concordance score on Korean-doctor prompts (mean 0.98, SD 0.82). However, this score did not reach the partially correct criterion of 1 on the 0 to 2-point scale. Clova X recorded the lowest mean score on English-patient prompts (mean 0.23, SD 0.63). Across both diagnostic categories, AGD specialists demonstrated the highest diagnostic accuracy (pulpal: 0.70; periapical: 0.65), with higher sensitivity but lower specificity than those exhibited by the other groups. ChatGPT also showed favorable performance among the LLMs, with accuracies of 0.65 (95% CI 0.55‐0.74) for pulpal disease and 0.57 (95% CI 0.47‐0.69) for periapical disease, which were comparable to those of AGD and endodontic residents.

**Conclusions:**

Under image-free clinical record review conditions, ChatGPT 4.0 showed relatively higher and more consistent performance in symptom screening and treatment planning compared to the other LLMs evaluated. However, its highest mean score of 0.98 (SD 0.82) did not reach the partially correct criterion of 1 on the 0 to 2-point scale. Hallucinations generated by LLMs and experience-dependent interpretation biases among human evaluators remain key challenges that require attention. Therefore, continuous clinical supervision and comprehensive user training are necessary for the safe and effective clinical application.

## Introduction

Accurate diagnosis of pulp pathologies plays a critical role in the success of root canal treatment, with the ultimate objective being the preservation of natural dentition [1,2]. In many clinical situations, root canal treatment is combined with periodontal and prosthodontic procedures, which complicates the process of diagnosis and treatment planning. A thorough evaluation of pulp and periapical tissue conditions, including the assessment of pulp vitality, remains essential before treatment initiation [2]. However, the pulp is surrounded by calcified tissue, which structurally limits direct examination [3]. Consequently, radiographic assessment remains an indispensable diagnostic modality for treatment planning and prognosis evaluation, playing a vital role in the objective confirmation of lesions [4,5].

The integration of imaging findings with clinical data often requires substantial time and expertise. Limited resources and time constraints frequently prevent clinicians from promptly incorporating the latest evidence or clinical guidelines into practice. Less experienced clinicians, in particular, are more vulnerable to diagnostic bias and errors, which may compromise diagnostic accuracy and clinical outcomes [2,6,7]. Recent advances in large language models (LLMs), which can process and generate human-like responses from unstructured patient records, have introduced new possibilities for supporting clinical assessment and treatment planning based on patient information [8,9].

LLMs can facilitate systematic clinical decision-making by integrating recent research findings and clinical guidelines. They may also provide value in complex cases by reconfirming overlooked information, reducing uncertainty, and supporting the development of individualized treatment strategies. This capability enables clinicians to refine diagnostic reasoning and design patient-specific treatment plans [10,11]. Therefore, evaluating the potential of LLMs as auxiliary tools to enhance symptom-based screening performance and treatment planning validity is a priority.

Most existing research on LLMs in conservative dentistry has focused on educational applications, such as responding to patient-related clinical questions or addressing multiple-choice examinations [12-16]. Furthermore, few studies have evaluated LLM performance using real-world clinical cases and compared model outputs with those of human evaluators. To address this gap, this study evaluated the clinical validity and relevance of LLM-generated symptom-based assessments and treatment planning responses using routine clinical records. This study examined how LLMs may function during the initial clinical assessment, where narrative clinical information guides early screening decisions, and compared their outputs with those of clinicians and senior students across different levels of clinical experience. In addition, we provide practical evidence on where LLM support may be clinically useful and where expert oversight remains essential. Therefore, this study aimed to provide clinically validated criteria for different applications of LLMs within the limited context of symptom-based screening in the field of dentistry.

## Methods

### Ethical Considerations

This retrospective study was approved by the institutional review board of the Dental Hospital of Yonsei University (IRB 2-2024-0075). All procedures were part of standard clinical care, and the committee waived the need for informed consent. To maintain privacy and confidentiality, all data were fully anonymized before analysis. This study did not involve participant compensation.

### Data Collection

This retrospective analysis included records of patients who visited the outpatient clinics of the Departments of Advanced General Dentistry (AGD) or Conservative Dentistry between January 2011 and December 2022. Eligible participants were adults aged 19 years or older, who had a recorded chief complaint at the initial visit, and were diagnosed with pulpal or periapical disease by a specialist. Among those who underwent pretreatment periapical radiography with corresponding clinical and radiographic records, 100 cases without previous endodontic treatment were randomly selected. The demographic and clinical characteristics of the included cases are summarized in Table 1. Chief complaints recorded in the clinical records reflect the patient-reported symptom history, whereas objective examination findings reflect the clinical status at the time of presentation.

**Table 1.** Demographic and clinical characteristics of the study cases.

Characteristics	Values (N=100)
Age (y), mean (SD)	57.22 (14.50)
<40	9 (9)
40‐59	44 (44)
≥60	47 (47)
Sex, n (%)	
Male	34 (34)
Female	66 (66)
Arch, n (%)	
Maxillary	46 (46)
Mandibular	54 (54)
Tooth type, n (%)	
Anterior tooth	12 (12)
Premolar	17 (17)
Molar	71 (71)
Symptoms, n (%)	
Pain on stimulus	47 (47)
Spontaneous pain	25 (25)
Asymptomatic	18 (18)
Tenderness on percussion	7 (7)
Other symptoms	3 (3)
Restoration status, n (%)	
Indirect restoration	45 (45)
None	30 (30)
Direct restoration	14 (14)
Bridge	11 (11)

### Study Design

Four LLMs were evaluated, namely ChatGPT (version 4.0, OpenAI Inc), Gemini (version 1.5 Pro, Google), Bing (Microsoft), and Clova X (version Hyper Clova X, Naver Corporation). All LLM queries were conducted between November 30, 2024, and December 7, 2024. Four prompt types were created by combining 2 variables—language (Korean vs English) and role (doctor vs patient): (1) Korean-doctor, (2) Korean-patient, (3) English-doctor, and (4) English-patient (Multimedia Appendix 1). All LLMs were queried using platform-default generation settings, without any manual adjustments. Clinical cases were provided in a narrative clinical record format, including patient age, sex, chief complaint, reason for visit, and clinical symptoms. Radiographic images were not directly provided to either the LLMs or the human evaluators. However, because radiographic interpretations are commonly documented in routine clinical information, summarized radiographic descriptors could be included within the chart text. Screening performance was compared with that of human evaluators using Korean-doctor prompts reflecting the clinical background of the evaluators. All treatment plan responses were reformatted into a narrative format before evaluation to ensure assessor blinding to the source of the response. English prompts were generated from the original Korean prompts using Google Translate (Google LLC) and reviewed by a domain expert for accuracy. To ensure reproducibility, prompt structure and generation settings were standardized. Persona prompts were adopted to reflect differences in clinical communication between clinicians and patients. A zero-shot prompting design was used to allow standardized evaluation without example-based guidance, thereby improving comparability across models and evaluator groups.

### Evaluator Groups

A conservative dentistry specialist with more than 20 years of clinical experience initially reviewed and selected suitable clinical cases for this study based on clinical data and radiographic assessments. The same specialist established the reference standard diagnosis using the full clinical and radiographic records and was blinded to the LLM-generated outputs and the diagnostic assessments of all evaluators. Twelve evaluators then independently assessed the cases using the same clinical information in the patient records provided to the LLMs. The evaluators included 3 AGD specialists with more than 5 years of experience, 3 AGD residents with more than 2 years of experience, 3 endodontic residents with more than 2 years of experience, and 3 senior dental students. Subsequently, the same evaluators assessed the clinical validity and relevance of the treatment plans generated by each LLM.

### Evaluation Criteria

Symptom-based screening accuracy was evaluated on a 0 to 2-point scale according to pathological concordance and the criteria of the *ICD-10* (*International Statistical Classification of Diseases*, *Tenth Revision*) [17]. Two points were assigned for diagnoses fully consistent with the reference standard, whereas 1 point was assigned for identification of the correct disease category with minor discrepancies in diagnostic specificity or terminology (eg, correct classification as pulpal disease with an imprecise subtype). No points were assigned for a complete mismatch (Multimedia Appendix 2). Because the doctor prompts requested *ICD-10*–based diagnoses while the reference standard used American Association of Endodontists terminology, differences in terminology between the 2 systems were handled within the existing assessment criteria, where responses identifying the correct disease category with a minor terminological discrepancy were scored as 1, and those fully consistent with the reference diagnosis were scored as 2. For screening performance analyses, the ordinal scores were binarized, with scores of 1 and 2 classified as positive and a score of 0 classified as negative. Diagnostic metrics were calculated using a one-versus-other scheme, in which pulpal cases (58/100) served as positives and periapical cases (42/100) as negatives for pulpal disease metrics, with the reverse applied for periapical disease metrics. For the human evaluator groups, responses from the 3 evaluators within each group were combined before calculating diagnostic metrics, resulting in an effective observation count of 174 per group for pulpal disease and 126 for periapical disease; 95% CIs reflect this aggregated count. The corresponding 2×2 contingency cell counts (true positive, false negative, false positive, and true negative) for all groups are provided in Multimedia Appendix 3. The clinical validity and relevance of LLM-generated treatment plans were assessed using a 5-point Likert scale [18,19]. Higher scores reflected greater clinical appropriateness and concordance with evidence-based practice. Evaluators independently rated the realism and evidence-based nature of each plan within a real-world clinical context; they were blinded to the LLM outputs (Multimedia Appendix 4).

### Statistical Analysis

To analyze differences between groups, the Kruskal-Wallis test was used, with post hoc pairwise comparisons conducted using the Dunn test with Bonferroni correction. Interrater agreement among the 4 evaluator groups was assessed using the intraclass correlation coefficient (ICC). Screening sensitivity, specificity, positive predictive value, negative predictive value, accuracy, and 95% CIs of the LLMs and clinicians were analyzed and compared for each disease. Diagnostic metrics and corresponding 95% CIs were calculated using the MedCalc Diagnostic Test Evaluation Calculator [20]. All statistical analyses were performed using SPSS (version 27, IBM Corp). Statistical significance was set at *P*<.05.

## Results

### Text-Based Screening Performance by Prompt Type

**Figure 1.**
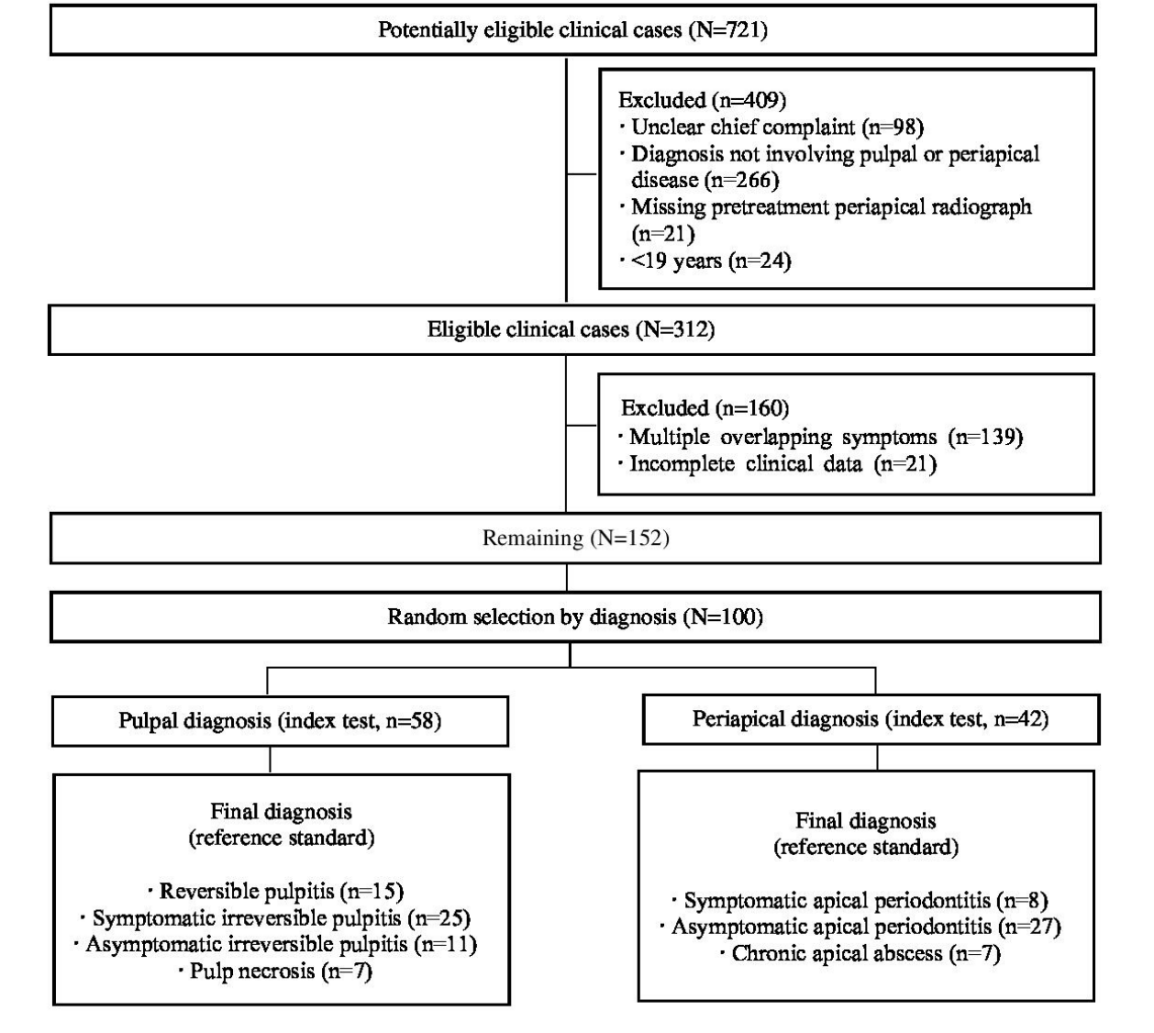
Flow diagram of case selection and diagnostic classification.

**Figure 2.**
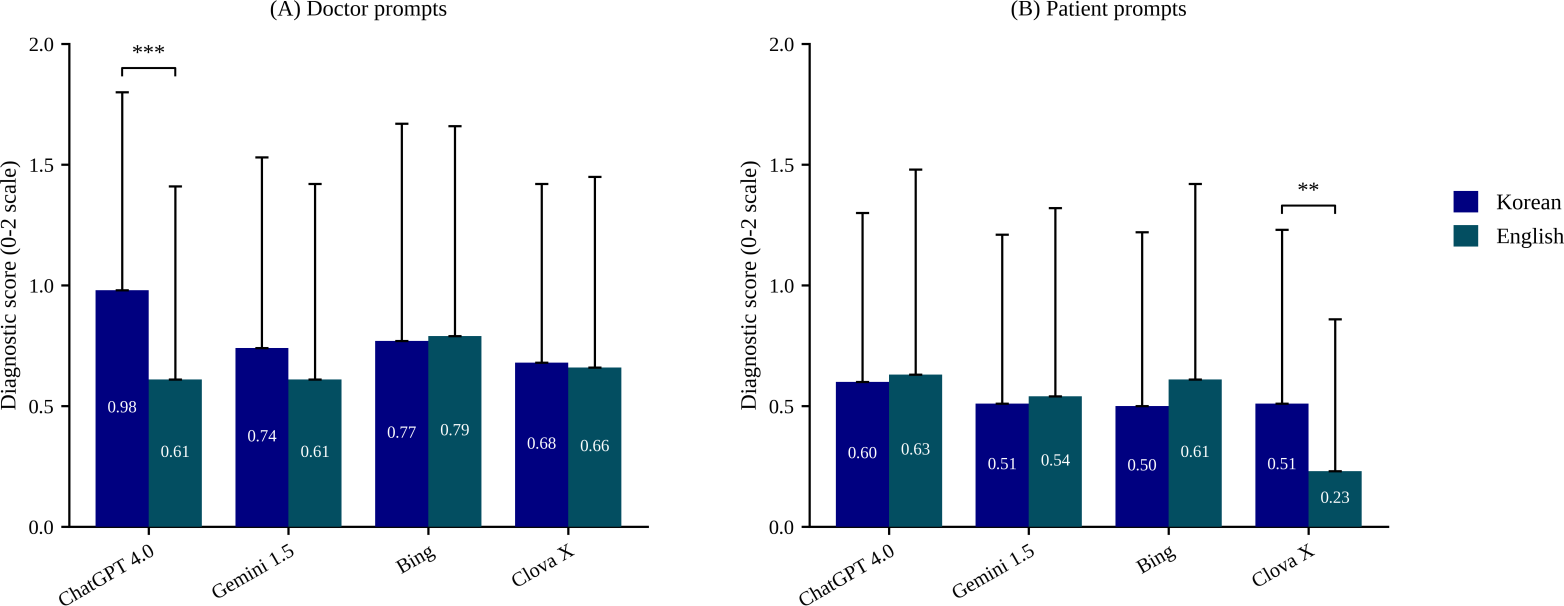
Diagnostic performance and consistency of 4 large language models across prompt types. Diagnostic scores (0 to 2-point scale) for ChatGPT 4.0, Gemini 1.5, Bing, and Clova X using (A) doctor and (B) patient prompts in Korean and English. Bars represent mean (SD). Statistically significant differences in diagnostic agreement were observed across languages within each model (**P<.01; *** *P*<.001).

The case selection and diagnostic classification process is shown in Figure 1. The text-based screening performance of LLMs, measured on a 0 to 2-point scale, was compared across prompt types (doctor vs patient) and languages (Korean vs English; Figure 2). ChatGPT achieved the highest concordance on Korean-doctor prompts (mean 0.98, SD 0.82), with a significant within-model difference between languages (*P*<.001). Conversely, Clova X recorded the lowest agreement on English-patient prompts (mean 0.23, SD 0.63), with a significant within-model difference (*P*=.004).

### Evaluation of Treatment Plan Relevance and Validity

**Figure 3.**
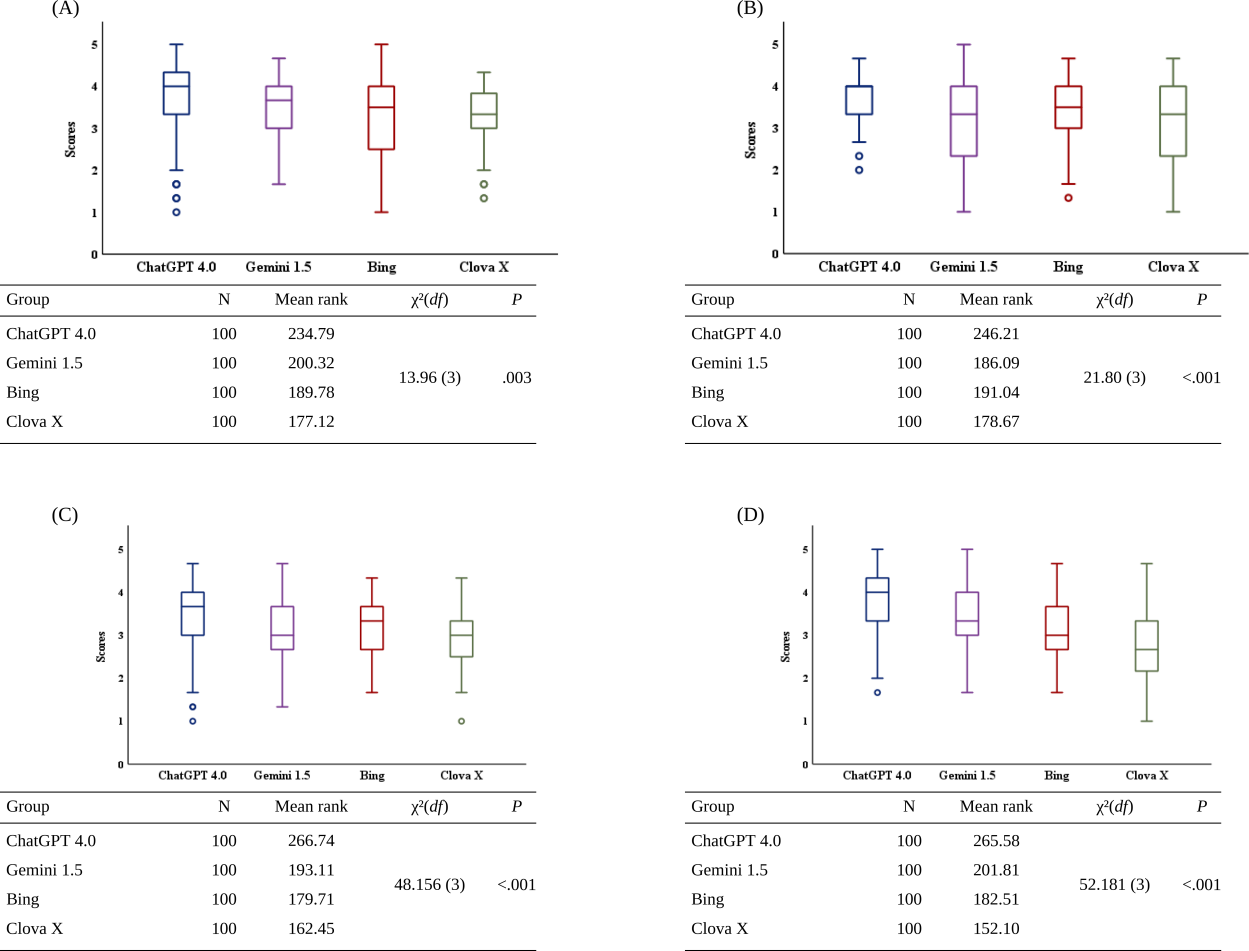
Relevance scores across large language models (LLMs) as evaluated by 4 human evaluator groups: (A) Advanced General Dentistry (AGD) specialists, (B) AGD residents, (C) endodontic residents, and (D) senior students. Box plots show median (IQR) and outliers of relevance scores on a 5-point Likert scale. Relevance reflects how directly LLM responses addressed the clinical questions posed in each prompt.

**Figure 4.**
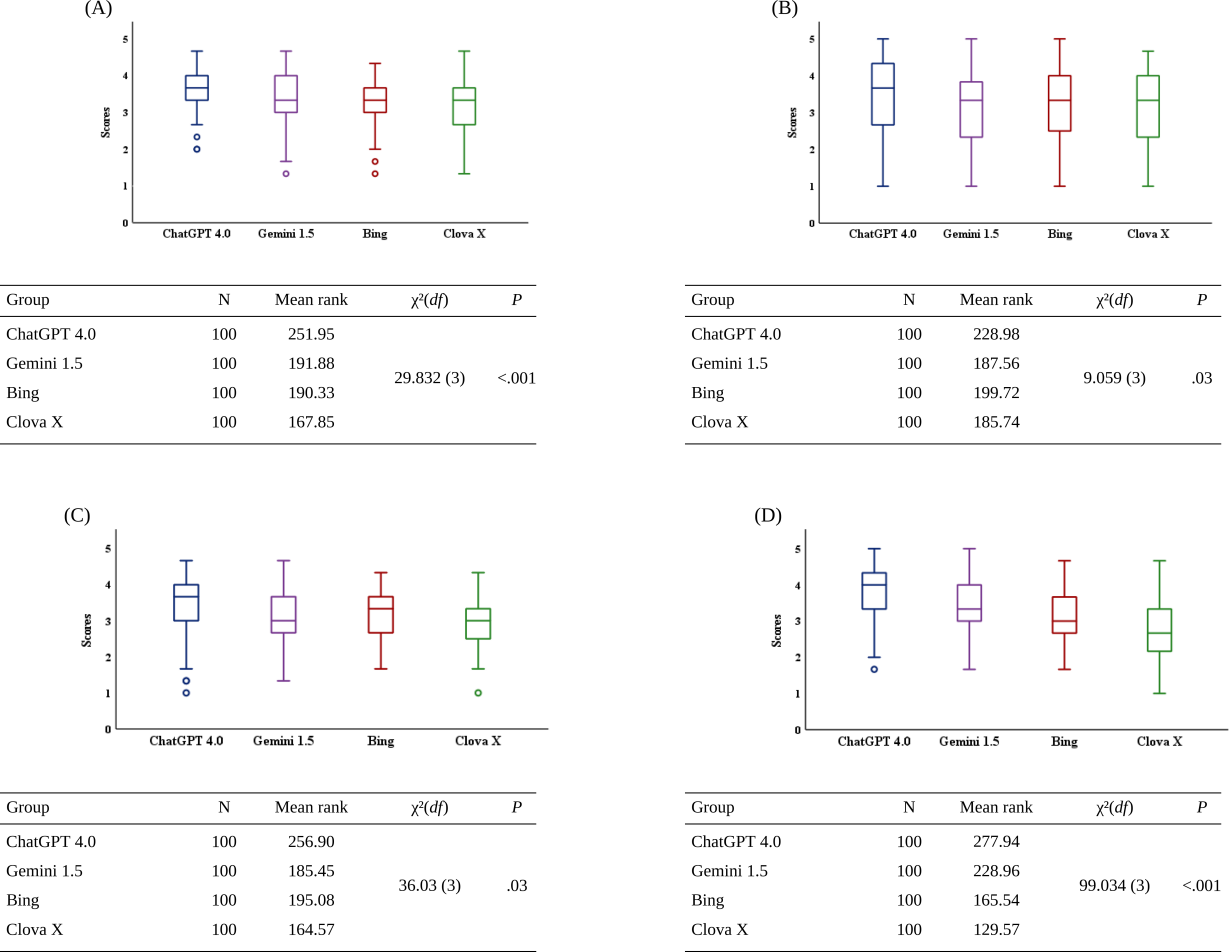
Validity scores across large language models (LLMs) as evaluated by 4 human evaluator groups: (A) Advanced General Dentistry (AGD) specialists, (B) AGD residents, (C) endodontic residents, and (D) senior students. Box plots show the median, IQR, and outliers of validity scores on a 5-point Likert scale. Validity reflects the clinical appropriateness and concordance of LLM responses with evidence-based diagnostic reasoning.

Across all evaluator groups, ChatGPT demonstrated higher mean ranks for treatment plan validity than other LLMs, whereas Clova X showed the lowest mean ranks (Figures 3 and 4). Statistically significant differences in both outcomes were observed among LLMs within each evaluator group.

### Comparison of Symptom-Based Assessment Scores

Symptom-based assessment scores on the 0 to 2-point scale were compared between LLMs and human evaluators using the Kruskal-Wallis test. AGD specialists achieved the highest mean rank (mean 1.10, SD 0.71; 466.81), followed by endodontic residents (mean 0.97, SD 0.79; 425.49), ChatGPT (mean 0.98, SD 0.82; 425.21), and AGD residents (mean 0.94, SD 0.78; 416.07). Senior dental students showed intermediate performance (mean 0.90, SD 0.71; 410.20), whereas Bing (mean 0.77, SD 0.90; 361.55), Gemini (mean 0.74, SD 0.79; 357.28), and Clova X (mean 0.68, SD 0.74; 341.40) demonstrated lower mean ranks (Table 2). There was a significant difference in symptom-based assessment scores among the groups (*χ*²_7_=25.75; *P*<.001).

**Table 2.** Comparison of symptom-based assessment scores across large language models and human evaluator groups using the Kruskal-Wallis test (N=100)^a^.

Group	Mean (SD)	Mean rank
AGD^b^ specialist	1.10 (0.71)	466.81^c^
Endodontic residents	0.97 (0.79)	425.49^d^
ChatGPT 4.0	0.98 (0.82)	425.21^d^
AGD residents	0.94 (0.78)	416.07^d^
Senior student	0.90 (0.71)	410.20^d^
Bing	0.77 (0.90)	361.55
Gemini 1.5	0.74 (0.79)	357.28
Clova X	0.68 (0.74)	341.40

a *χ*²_7_=25.75; *P*<.001.

b AGD: Advanced General Dentistry.

c Mean ranks of AGD specialist are significantly different from Bing, Gemini 1.5, and Clova X.

d Mean ranks of endodontic residents, ChatGPT 4.0, AGD residents, and senior students are not significantly different from either AGD specialists or Bing, Gemini 1.5, and Clova X based on Dunn post hoc test with Bonferroni correction (*P*>.05).

Interrater agreement among the 4 evaluator groups, measured by the ICC, was lowest for AGD specialists (ICC 0.579, 95% CI 0.413‐0.704; *P*<.001). Moderate agreement was observed for AGD residents (ICC 0.774, 95% CI 0.685‐0.841; *P*<.001), endodontic residents (ICC 0.793, 95% CI 0.711‐0.854; *P*<.001), and senior dental students (ICC 0.750, 95% CI 0.651‐0.824; *P*<.001; Table 3). Post hoc pairwise comparisons using the Dunn test with Bonferroni correction showed that AGD specialists scored significantly higher than Clova X, Gemini, and Bing (Multimedia Appendix 5).

**Table 3.** Interrater reliability of diagnostic evaluations across evaluator groups.

Evaluator group	ICC^a^, average measure (95% CI)	*F* value (*df*)	*P* value
AGD^b^ specialists	0.579 (0.413‐0.704)	2.377 (99, 198)	<.001
AGD residents	0.774 (0.685‐0.841)	4.431 (99, 198)	<.001
Endodontic residents	0.793 (0.711‐0.854)	4.830 (99, 198)	<.001
Senior dental students	0.750 (0.651‐0.824)	3.995 (99, 198)	<.001

a ICC: intraclass correlation coefficient.

b AGD: Advanced General Dentistry.

### Comparative Diagnostic Metrics

Tables 4 and 5 present the diagnostic metrics for pulpal and periapical diseases, respectively. AGD specialists showed the highest diagnostic accuracy among all groups for both pulpal (0.70) and periapical diseases (0.65). Among the LLMs, ChatGPT demonstrated the highest accuracy for pulpal disease (0.65, 95% CI 0.55‐0.74), whereas Clova X showed the lowest accuracy (0.47, 95% CI 0.37‐0.57). For periapical disease, the overall diagnostic performance of LLMs was lower, with ChatGPT achieving an accuracy of 0.57 (95% CI 0.47‐0.69).

**Table 4.** Diagnostic metrics (accuracy, sensitivity, and specificity with 95% CI) of large language models (LLMs) and human evaluators for pulpal disease (n=58)^a^.

Diagnostic metrics	ChatGPT 4.0	Gemini 1.5 Pro	Bing	Clova X	AGD^b^ specialist	AGD residents	Endodontic residents	Senior students
Sensitivity	0.60 (0.47‐0.73)	0.55 (0.42‐0.68)	0.45 (0.32‐0.58)	0.40 (0.27‐0.53)	0.79 (0.72‐0.85)	0.69 (0.62‐0.76)	0.71 (0.64‐0.78)	0.51 (0.43‐0.59)
Specificity	0.71 (0.55‐0.84)	0.55 (0.39‐0.70)	0.69 (0.53‐0.82)	0.57 (0.41‐0.72)	0.57 (0.48‐0.66)	0.65 (0.56‐0.73)	0.67 (0.58‐0.75)	0.61 (0.52‐0.70)
PPV^c^	0.74 (0.63‐0.83)	0.63 (0.53‐0.71)	0.67 (0.54‐0.77)	0.56 (0.44‐0.67)	0.72 (0.67‐0.76)	0.73 (0.68‐0.78)	0.75 (0.69‐0.79)	0.64 (0.58‐0.70)
NPV^d^	0.57 (0.48‐0.68)	0.47 (0.37‐0.57)	0.48 (0.40‐0.55)	0.41 (0.33‐0.49)	0.66 (0.58‐0.73)	0.60 (0.54‐0.66)	0.63 (0.56‐0.69)	0.48 (0.42‐0.52)
Accuracy	0.65 (0.55‐0.74)	0.55 (0.45‐0.65)	0.55 (0.45‐0.65)	0.47 (0.37‐0.57)	0.70 (0.64‐0.75)	0.67 (0.62‐0.73)	0.69 (0.64‐0.75)	0.55 (0.50‐0.61)

a Human evaluator metrics were calculated by combining responses across 3 evaluators per group (effective n=174); LLM metrics reflect single-pass evaluations (n=58).

b AGD: Advanced General Dentistry.

c PPV: positive predictive value.

d NPV: negative predictive value.

**Table 5.** Diagnostic metrics (accuracy, sensitivity, specificity with 95% CI) of large language models (LLMs) and human evaluators for periapical disease (n=42)^a^.

Diagnostic metrics	ChatGPT 4.0	Gemini 1.5 Pro	Bing	Clova X	AGD^b^ specialist	AGD residents	Endodontic residents	Senior students
Sensitivity	0.43 (0.28‐0.59)	0.29 (0.16‐0.45)	0.43 (0.28‐0.59)	0.24 (0.12‐0.39)	0.64 (0.55‐0.73)	0.45 (0.36‐0.54)	0.52 (0.43‐0.61)	0.40 (0.31‐0.49)
Specificity	0.67 (0.54‐0.79)	0.62 (0.48‐0.74)	0.59 (0.45‐0.71)	0.72 (0.59‐0.83)	0.66 (0.59‐0.73)	0.64 (0.56‐0.71)	0.62 (0.54-0.69)	0.58 (0.50‐0.65)
PPV^c^	0.49 (0.36‐0.61)	0.35 (0.23‐0.49)	0.43 (0.32‐0.54)	0.38 (0.24‐0.55)	0.58 (0.52‐0.64)	0.48 (0.41‐0.54)	0.50 (0.44-0.56)	0.41 (0.34‐0.47)
NPV^d^	0.62 (0.54‐0.69)	0.55 (0.48‐0.61)	0.59 (0.50‐0.67)	0.57 (0.51‐0.62)	0.72 (0.66‐0.77)	0.62 (0.57‐0.66)	0.64 (0.59-0.69)	0.57 (0.52‐0.62)
Accuracy	0.57 (0.47‐0.69)	0.48 (0.38‐0.58)	0.52 (0.42‐0.62)	0.52 (0.42‐0.62)	0.65 (0.60‐0.71)	0.56 (0.50‐0.62)	0.58 (0.52-0.64)	0.50 (0.45‐0.56)

a Human evaluator metrics were calculated by combining responses across 3 evaluators per group (effective n=126). LLM metrics reflect single-pass evaluations (n=42).

b AGD: Advanced General Dentistry.

c PPV: positive predictive value.

d NPV: negative predictive value.

### Distribution of Diagnostic Scores

**Figure 5.**
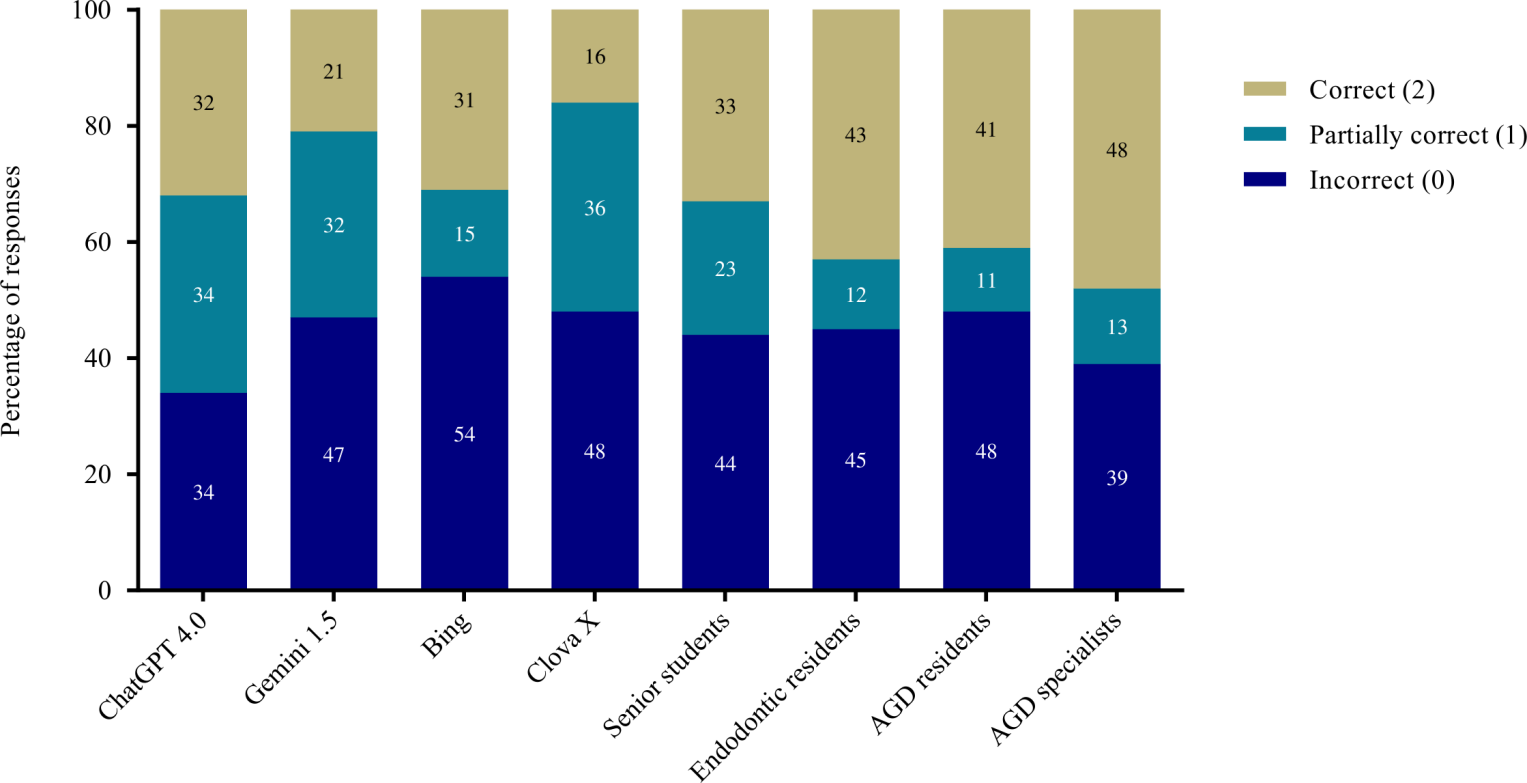
Distribution of diagnostic accuracy scores among large language models (LLMs) and human evaluator groups. Stacked bar graphs show the proportion (%) of diagnostic accuracy scores (0=“incorrect,” 1=“partially correct,” and 2=“correct”) for 4 LLMs and human evaluator groups. Among all groups, Advanced General Dentistry (AGD) specialists demonstrated the highest proportion of correct responses, followed by endodontic residents and AGD residents. ChatGPT 4.0 outperformed Gemini, Bing, and Clova X in diagnostic accuracy.

Distribution analysis of diagnostic scores demonstrated that AGD specialists had the highest proportion of correct diagnoses at 48% (48/100), followed by endodontic residents at 43% (43/100), AGD residents at 41% (41/100), and senior dental students at 33% (33/100; Figure 5). Among the LLMs, ChatGPT produced the highest proportion of correct responses at 32% (32/100), surpassing that of senior dental students but remaining lower than that of specialists and residents. Regarding incorrect responses, Bing had the highest proportion at 54% (54/100), followed by Clova X at 48% (48/100). An exploratory subgroup analysis comparing patients aged younger than 60 years and 60 years or older demonstrated no significant difference in diagnostic accuracy between the groups (*P*=.051).

## Discussion

### Principal Findings

This study compared the performance of LLM-generated outputs with that of clinicians and senior dental students, evaluating the clinical validity and relevance of LLM-derived treatment plans. AGD specialists achieved the highest symptom-based screening accuracy and sensitivity. Among the LLMs, ChatGPT consistently showed the highest performance, with screening accuracy and diagnostic metrics comparable to those of AGD and endodontic residents. However, its relatively low sensitivity for pulpal disease diagnosis represents an important safety limitation, indicating that LLM-generated outputs should not be used for independent definitive diagnosis without expert supervision.

The 0 to 2 points scoring system enabled standardized comparison across LLMs and human evaluators. However, partially correct responses included a broad range of diagnostic differences, from minor imprecision to clinically meaningful misclassification. Therefore, the findings should be interpreted with caution, as this simplified scoring may not fully reflect the clinical impact of specific diagnostic errors. Although AGD specialists demonstrated high screening accuracy, their interrater agreement was relatively low, likely reflecting variability in expert interpretation of ambiguous or incomplete clinical information. This variability does not indicate poorer diagnostic ability but highlights the complexity of decision-making under limited clinical information [21,22]. LLMs did not achieve specialist-level performance; however, ChatGPT showed potential as a supportive tool for less experienced clinicians. Post hoc pairwise analysis further showed that AGD specialists scored significantly higher than Clova X, Gemini, and Bing. In contrast, no statistically significant difference was detected between ChatGPT and any of the human evaluator groups. These results are consistent with our overall finding that ChatGPT performed relatively better than the other LLMs evaluated, while overall performance remained modest. Nonetheless, hallucinations were observed, including fabricated clinical details and treatment plans inconsistent with established guidelines (Multimedia Appendix 6).

### Comparisons With Prior Work

Although recent studies have investigated the use of LLMs in conservative dentistry, most have focused on educational settings, patient inquiries, or multiple-choice examinations [12-16] (Table 6). In contrast, this study used real-world data under multiple prompting scenarios. Zero-shot prompts and personas were applied to reflect practical clinical use, where optimized prompting cannot be assumed [23].

**Table 6.** Comparison of this study with prior large language model (LLM) studies in dentistry.

Study	Data source	Clinical task	Evaluators	Outcomes assessed
Abdulrab et al [12], 2025	Exam-style questions	Knowledge assessment	Students	Accuracy
Aljamani et al [15], 2025	Patient inquiries	Patient information support	None	Response quality
Büker and Mercan [13], 2025	Patient inquiries	Patient information quality assessment	None	Readability, accuracy, and quality
Jalali et al [14], 2025	Board-style exam questions	Board exam performance	None	Accuracy
Arılı Öztürk et al [16], 2025	Endodontic exam-style questions	Knowledge assessment in endodontics	None	Answer accuracy
This study	Text-based clinical records (image-free)	Symptom-based diagnostic screening and treatment planning	Specialists, residents, and students	Diagnostic accuracy, treatment plan validity, and interrater reliability

Outputs varied substantially by language and prompt type, underscoring the importance of prompt design in clinical contexts [24,25]. Consistent with prior medical studies, role-specific prompts improved clinical relevance, whereas vague prompts reduced consistency [26,27]. The student group rated the LLM-generated treatment plans more positively than experienced clinicians, likely reflecting differences in error recognition ability based on clinical experience. Tangadulrat et al [28] reported similar findings in medicine, where medical students tended to trust ChatGPT’s results more than physicians did. These results suggest that clinical experience is critical for evaluating AI-generated outputs. As summarized in Table 6, this study differs from prior work.

Recent advances in multimodal LLMs, such as GPT-4V and Gemini Pro Vision, demonstrate improved diagnostic performance when radiographic images are incorporated into diagnostic assessments [29,30]. In this study, radiographic images were not directly reviewed, although summarized radiographic findings were available in clinical records. Given the indispensable role of radiographic examination in endodontic diagnosis, limited access to image interpretation may partly explain the lower sensitivity observed for LLMs compared with specialists. Incorporating radiographic image analysis into future dental AI systems may improve diagnostic performance in clinical practice.

Previous studies in conservative dentistry have mainly examined the use of LLMs in educational contexts and multiple-choice examinations. Previous studies by Arılı Öztürk et al [16], Abdulrab et al [12], and Künzle and Paris [31] consistently demonstrated that more advanced models, such as ChatGPT-4 and ChatGPT-4o, showed higher accuracy and stability than earlier versions or other models. Durmazpinar and Ekmekci [32] found that ChatGPT-4o outperformed students in multiple-choice questions. In addition, Nguyen et al [33] observed that ChatGPT performed well in text-based questions but showed lower accuracy in image-based questions.

Similar findings have been reported in studies addressing patients’ clinical inquiries. Aljamani et al [15], Baris and Baris [34], and Büker and Mercan [13] showed that several models achieved high accuracy and quality in answering patients’ questions related to endodontic treatment. However, these studies consistently highlighted important limitations, including higher reading levels, variability in reliability, and the potential for misinterpretation without expert supervision.

### Limitations

This study had several limitations. First, a zero-shot prompting design was used, and follow-up questions were intentionally excluded, which limited the evaluation of iterative clinical reasoning [35]. Consequently, this study could not assess whether multiturn prompting, which allows iterative clarification and the inclusion of additional information, would alter diagnostic accuracy or reasoning performance.

Second, although the English prompts were reviewed by a domain expert, translation nuances may have influenced model interpretation. Notably, *per* (*+*) was used instead of the standard English shorthand for percussion sensitivity, and *conservative dentist* was used instead of the internationally recognized term *endodontist*. The study was also limited to English and Korean prompts, which may have restricted the generalizability of the findings to other linguistic or cultural contexts.

Third, the analysis relied on clinical record information. Radiographic images were not directly reviewed by LLMs or human evaluators, although summarized radiographic interpretations were available in routine documentation. Diagnostic performance may, therefore, have been underestimated. Accordingly, direct comparability between the reference standard and test conditions was limited. Therefore, it remains unclear whether diagnostic failures reflect limited reasoning capability or simply reflect missing diagnostic data. A single specialist determined the reference diagnosis, and pre-2017 cases were not reclassified under updated criteria. In addition, no formal sample size calculation was performed, as the sample size was determined by the available eligible cases. This may have limited statistical power for some comparisons. Furthermore, 139 of 312 eligible cases were excluded due to multiple concurrent symptoms, which may have resulted in a less clinically diverse set of included cases. Therefore, the diagnostic performance of both LLMs and human evaluators may have been higher than that observed in routine clinical practice, where patients frequently present with overlapping symptoms.

Fourth, doctor and patient prompts differed in both persona and the amount of clinical information provided, as doctor prompts included objective examination findings and radiographic descriptors, while patient prompts relied solely on subjective symptoms. Therefore, it remains unclear whether the observed performance differences were attributable to persona framing or to the difference in available clinical data. Additionally, the doctor prompts requested *ICD-10*–based diagnoses, while the reference standard used AAE terminology. Where the 2 classification systems do not correspond one-to-one in certain categories, model responses may have been more likely to receive a score of 1 rather than 2, potentially underestimating performance under doctor-prompt conditions.

Fifth, the Kruskal-Wallis test assumes independent samples; however, because all groups assessed the identical set of 100 clinical cases, this assumption is not fully satisfied. This limitation should be considered when interpreting both Table 2 and the post hoc pairwise comparisons in Multimedia Appendix 1. Additionally, the one-versus-other classification scheme assigns each case to a single disease category. It may not fully capture clinically linked dual diagnoses, potentially classifying biologically comprehensive responses as false positives. Furthermore, pooling responses across 3 evaluators within each group may constitute pseudoreplication, potentially narrowing the reported 95% CIs in Tables 4 and 5. In addition, scores of 1 and 2 were both classified as positive in the binary diagnostic performance analysis. Although this approach was intended to reflect clinically meaningful partial agreement, grouping partially correct diagnoses as positive outcomes may have inflated the reported sensitivity and accuracy metrics (Multimedia Appendix 7).

Finally, hallucination and interpretation biases were observed, but qualitative error classification was not performed. Future studies incorporating qualitative analyses and multimodal inputs are warranted for better characterization of safety risks and improved clinical applicability. Despite these limitations, this study provides a comprehensive evaluation using real-world clinical data under diverse prompting conditions.

### Future Outlook

Future research should evaluate multimodal LLMs integrating text and imaging data under identical clinical conditions. In addition, advanced prompting strategies—including chain-of-thought and few-shot prompting—should be examined to enhance structured clinical reasoning and diagnostic accuracy. Approaches that constrain model outputs using established clinical guidelines or retrieval-augmented generation may help mitigate hallucinations. Moreover, interactive conversational frameworks that enable clarification of missing information may also enhance clinical reasoning and reliability. From a clinical implementation perspective, a human-in-the-loop workflow may be more appropriate than independent model use. Given the relatively high specificity but limited sensitivity observed, LLMs may be better suited for generating differential diagnosis lists rather than for definitive screening. In this workflow, clinicians review model-generated suggestions and integrate them with clinical examination and radiographic findings before making final decisions.

### Conclusions

Compared to other LLMs, ChatGPT provides more stable and reliable responses in endodontic diagnosis, demonstrating potential as a supportive tool for clinicians. However, limitations such as hallucinations, low sensitivity, and variability depending on user expertise remain significant challenges to its use as an independent diagnostic aid. For safe and effective clinical application, the development of standardized prompt templates, the implementation of comprehensive user training, and continued clinical supervision will be essential.

## Supplementary material

10.2196/86145 Multimedia Appendix 1 Prompt templates used for the 4 prompt types (Korean-doctor, Korean-patient, English-doctor, and English-patient) administered to all large language models.

10.2196/86145 Multimedia Appendix 2 Evaluation criteria for symptom-based screening performance using the 0 to 2-point concordance scale.

10.2196/86145 Multimedia Appendix 3 Pooled 2×2 contingency cell counts underlying the diagnostic metrics reported in Multimedia Appendix 4.

10.2196/86145 Multimedia Appendix 4 Evaluation criteria for treatment plan validity and relevance using the 5-point Likert scale.

10.2196/86145 Multimedia Appendix 5 Post hoc pairwise comparisons of symptom-based assessment scores across large language models and human evaluator groups using the Dunn test with Bonferroni correction (all 28 pairwise comparisons).

10.2196/86145 Multimedia Appendix 6 Representative examples of hallucinated and clinically inconsistent responses across the 4 large language models evaluated.

10.2196/86145 Multimedia Appendix 7 Strict diagnostic accuracy metrics (sensitivity, specificity, positive predictive value, negative predictive value, and accuracy).

## Abbreviations

AGD: Advanced General Dentistry
ICC: intraclass correlation coefficient
*ICD-10*: *International Statistical Classification of Diseases, Tenth Revision*
LLM: large language model
