# Supplementary material for: Large Language Models for Endodontic Symptom Assessment and Treatment Planning Using Image-Free Clinical Records: Comparative Evaluation Study
Source: JMIR Med Inform. 2026 Jul 24;14:e86145. doi: 10.2196/86145 (PMC13399569; doi:10.2196/86145)
Supplement: Multimedia Appendix 1 [file medinform-v14-e86145-s001.docx]

| **Supplemental Table 1a. Verbatim Korean-doctor Prompt used for Large Language Model Evaluation** |
| --- |
| 지금부터 너가 대학병원에서 근무하는 보존과 전문의라는 가정하에 이 환자에 대해서 ICD-10 Code를 기준으로 진단하고 어떻게 치료 할 지 설명해줘.  C.C. 44세 남자환자로 한달전부터 왼쪽 어금니가(#27) 약간 불편했었다. 그런데 어제밤부터 왼쪽 제일 뒤 어금니가 많이 아팠고, 밤부터는 잠도 못잤다 (onset : 1달 전) 동네치과에서 보존과를 추천해주셨다. -자발통(-) 저작통(+) 냉온자극(-) -타이레놀 1회 복용시 증상 개선됨 curettage (2017년 8월, d/t* perio) per(+) bite(+) with PAR. |
| *d/t, due to. |

| **Supplemental Table 1b. Verbatim Korean-patient Prompt used for Large Language Model Evaluation** |
| --- |
| 나는 44살 남자이고 지금 치아가 안좋아서 치과에 가야할 것 같은데 한달전부터 왼쪽 어금니가 약간 불편했었는데 어제밤부터 왼쪽 제일 뒤 어금니가 많이 아팠고, 밤부터는 잠도 못잤어. 씹을때 통증이 있고 차가운거나 뜨거운거에는 특별히 증상은 없어. 동네치과에서 보존과를 추천해줘서 가보려고하는데, 지금 상태에서 가장 가능성 높은 진단이 뭔지 알려줘. |

| **Supplemental Table 1c. Verbatim English-doctor Prompt used for Large Language Model Evaluation** |
| --- |
| From now on, assuming you are a conservative dentist working at a university hospital, diagnose this patient based on the ICD-10 code and explain the treatment approach.  C.C., a 44-year-old male, has had some discomfort in his left molar (#27) for the past month. However, starting last night, his left posterior molar has been in severe pain, and he hasn't been able to sleep since. (Onset: 1 month ago) The local dental clinic recommended a conservative dentist. - Spontaneous pain (-), pain with chewing (+), cold and hot stimulation (-) - Symptoms improved with a single dose of Tylenol. Curettage (August 2017, d/t perio), per (+), bite (+) with PAR. |

| **Supplemental Table 1d. Verbatim English-patient Prompt used for Large Language Model Evaluation** |
| --- |
| I'm a 44-year-old male with a bad tooth and need to see a dentist. I've had some discomfort in my left molar for the past month. Starting last night, my left posterior molar has been in severe pain, and I haven't been able to sleep since. There is pain when chewing, but there are no special symptoms when it comes to cold or hot things. The local dental clinic recommended a conservative dentist. Just tell me the most likely diagnosis at this point. |
