# Supplementary material for: Large Language Models for Endodontic Symptom Assessment and Treatment Planning Using Image-Free Clinical Records: Comparative Evaluation Study
Source: JMIR Med Inform. 2026 Jul 24;14:e86145. doi: 10.2196/86145 (PMC13399569; doi:10.2196/86145)
Supplement: Multimedia Appendix 2 [file medinform-v14-e86145-s002.docx]

| **Supplemental Table 2. Standardized Assessment Criteria for Diagnosis** | |  |
| --- | --- | --- |
| **Standard** | | **Points** |
| Incorrect | The diagnosis is entirely incorrect, either representing a disease with a different pathological mechanism or not corresponding to the clinical diagnosis | 0 |
| Partially correct | The diagnosis identifies the correct disease category but differs in specific details, such as an imprecise subtype or minor terminological discrepancy | 1 |
| Correct | The diagnosis is fully consistent with the reference standard and belongs to the category defined by the ICD-10 classification | 2 |
