# Supplementary material for: Large Language Models for Endodontic Symptom Assessment and Treatment Planning Using Image-Free Clinical Records: Comparative Evaluation Study
Source: JMIR Med Inform. 2026 Jul 24;14:e86145. doi: 10.2196/86145 (PMC13399569; doi:10.2196/86145)
Supplement: Multimedia Appendix 3 [file medinform-v14-e86145-s003.docx]

| **Supplemental Table 3.** Pooled 2×2 contingency table cell counts underlying the diagnostic metrics reported in Tables 4a and 4b | | | | | |
| --- | --- | --- | --- | --- | --- |
| **Table 3a. Pulpal Disease** | | | | | |
| **Group** | **TP^a^** | **FN^b^** | **FP^c^** | **TN^d^** | **Total** |
| **LLMs^e^(n=100)** | | | | | |
| ChatGPT 4.0 | 35 | 23 | 12 | 30 | 100 |
| Gemini 1.5 Pro | 32 | 26 | 19 | 23 | 100 |
| Bing | 26 | 32 | 13 | 29 | 100 |
| Clova X | 23 | 35 | 18 | 24 | 100 |
| **Human (n=300)** | | | | | |
| AGD^f^ Specialist | 137 | 37 | 54 | 72 | 300 |
| AGD Residents | 120 | 54 | 44 | 82 | 300 |
| Endodontic Residents | 124 | 50 | 42 | 84 | 300 |
| Senior Students | 89 | 85 | 49 | 77 | 300 |
| ^a^TP, true positive; ^b^FN, false negative; ^c^FP, false positive; ^d^TN, true negative; ; ^e^LLM, large language model; ^f^AGD, Advanced General Dentistry.  For human evaluator groups, responses from the three evaluators per group were combined before computing the contingency cell counts. | | | | | |

| **Table 3b. Periapical Disease** | | | | | | | | | |
| --- | --- | --- | --- | --- | --- | --- | --- | --- | --- |
| **Group** | **TP^a^** | | **FN^b^** | | **FP^c^** | | **TN^d^** | | **Total** |
| **LLMs^e^(n=100)** | | | | | | | | | |
| ChatGPT 4.0 | 18 | | 24 | | 19 | | 39 | | 100 |
| Gemini 1.5 Pro | 12 | | 30 | | 22 | | 36 | | 100 |
| Bing | 18 | | 24 | | 24 | | 34 | | 100 |
| Clova X | 10 | | 32 | | 16 | | 42 | | 100 |
| **Human (n=300)** | | | | | | | | | |
| AGD^f^ Specialist | 81 | | 45 | | 59 | | 115 | | 300 |
| AGD Residents | 57 | | 69 | | 63 | | 111 | | 300 |
| Endodontic Residents | 66 | | 60 | | 66 | | 108 | | 300 |
| Senior Students | 50 | 76 | | 73 | | 101 | | 300 | |
| ^a^TP, true positive; ^b^FN, false negative; ^c^FP, false positive; ^d^TN, true negative; ; ^e^LLM, large language model; ^f^AGD, Advanced General Dentistry.  For human evaluator groups, responses from the three evaluators per group were combined before computing the contingency cell counts. | | | | | | | | | |
