# Supplementary material for: Large Language Models for Endodontic Symptom Assessment and Treatment Planning Using Image-Free Clinical Records: Comparative Evaluation Study
Source: JMIR Med Inform. 2026 Jul 24;14:e86145. doi: 10.2196/86145 (PMC13399569; doi:10.2196/86145)
Supplement: Multimedia Appendix 4 [file medinform-v14-e86145-s004.docx]

| **Supplemental Table 3. Criteria for evaluating clinical validity and relevance** | | |
| --- | --- | --- |
| **Evaluation List** | | **Scores** |
| Highly Inaccurate | Diagnosis is entirely incorrect or not clinically relevant | 1 |
| Moderately Inaccurate | Diagnosis is partially incorrect or lacks clinical significance | 2 |
| Partially Accurate | Diagnosis is partially accurate but lacks completeness (e.g., correct disease category but insufficient specificity) | 3 |
| Substantially Accurate | Diagnosis is substantially accurate with minor discrepancies | 4 |
| Highly Accurate | Diagnosis is highly accurate and nearly identical to the reference standard | 5 |
