# Supplementary material for: Large Language Models for Endodontic Symptom Assessment and Treatment Planning Using Image-Free Clinical Records: Comparative Evaluation Study
Source: JMIR Med Inform. 2026 Jul 24;14:e86145. doi: 10.2196/86145 (PMC13399569; doi:10.2196/86145)
Supplement: Multimedia Appendix 5 [file medinform-v14-e86145-s005.docx]

| **Supplemental Table 5.** Post-hoc pairwise comparisons of symptom-based assessment scores across large language models and human evaluator groups using Dunn's test with Bonferroni correction (all 28 pairwise comparisons) | | | | |
| --- | --- | --- | --- | --- |
| **Sample 1 – Sample 2** | **Test Statistic** | ***Z*** | ***P*** | **Adjusted *P*ᵃ** |
| ^b^AGD Specialist – Endodontic Residents | 41.315 | 1.308 | .191 | 1.000 |
| AGD Specialist – ChatGPT 4.0 | 41.595 | 1.317 | .188 | 1.000 |
| AGD Specialist – AGD Residents | 50.735 | 1.606 | .108 | 1.000 |
| AGD Specialist – Senior Students | 56.605 | 1.792 | .073 | 1.000 |
| AGD Specialist – Bing | 105.260 | 3.332 | <.001 | **.024** |
| AGD Specialist – Gemini 1.5 | 109.525 | 3.467 | <.001 | **.015** |
| AGD Specialist – Clova X | 125.405 | 3.970 | <.001 | **.002** |
| Endodontic Residents – ChatGPT 4.0 | 0.280 | 0.009 | .993 | 1.000 |
| Endodontic Residents – AGD Residents | 9.420 | 0.298 | .766 | 1.000 |
| Endodontic Residents – Senior Students | 15.290 | 0.484 | .628 | 1.000 |
| Endodontic Residents – Bing | 63.945 | 2.024 | .043 | 1.000 |
| Endodontic Residents – Gemini 1.5 | 68.210 | 2.159 | .031 | .863 |
| Endodontic Residents – Clova X | 84.090 | 2.662 | .008 | .218 |
| ChatGPT 4.0 – AGD Residents | 9.140 | 0.289 | .772 | 1.000 |
| ChatGPT 4.0 – Senior Students | 15.010 | 0.475 | .635 | 1.000 |
| ChatGPT 4.0 – Bing | 63.665 | 2.015 | .044 | 1.000 |
| ChatGPT 4.0 – Gemini 1.5 | 67.930 | 2.150 | .032 | .883 |
| ChatGPT 4.0 – Clova X | 83.810 | 2.653 | .008 | .223 |
| AGD Residents – Senior Students | 5.870 | 0.186 | .853 | 1.000 |
| AGD Residents – Bing | 54.525 | 1.726 | .084 | 1.000 |
| AGD Residents – Gemini 1.5 | 58.790 | 1.861 | .063 | 1.000 |
| AGD Residents – Clova X | 74.670 | 2.364 | .018 | .507 |
| Senior Students – Bing | 48.655 | 1.540 | .124 | 1.000 |
| Senior Students – Gemini 1.5 | 52.920 | 1.675 | .094 | 1.000 |
| Senior Students – Clova X | 68.800 | 2.178 | .029 | .824 |
| Bing – Gemini 1.5 | 4.265 | 0.135 | .893 | 1.000 |
| Bing – Clova X | 20.145 | 0.638 | .524 | 1.000 |
| Gemini 1.5 – Clova X | 15.880 | 0.503 | .615 | 1.000 |
| ᵃAdjusted *P* values are Bonferroni-corrected. All pairwise comparisons share a common standard error of 31.590. Test statistics are reported as absolute values, with comparisons listed in the order of Table 2.  ^b^AGD, Advanced General Dentistry. | | | | |
