# Supplementary material for: Large Language Models for Endodontic Symptom Assessment and Treatment Planning Using Image-Free Clinical Records: Comparative Evaluation Study
Source: JMIR Med Inform. 2026 Jul 24;14:e86145. doi: 10.2196/86145 (PMC13399569; doi:10.2196/86145)
Supplement: Multimedia Appendix 6 [file medinform-v14-e86145-s006.docx]

| **Supplemental Table 6. Representative examples of hallucinated clinically inconsistent LLM responses across the four LLMs evaluated.** | | | |
| --- | --- | --- | --- |
| Model | Example response | Error type | Clinical implication |
| ChatGPT 4.0 | This condition appears to have progressed chronically, and the patient likely experiences minimal pain. | Absence of pain inferred despite no explicit report of pain severity | The model generated a clinical assumption not supported by the case description. |
| Gemini 1.5 Pro | The patient may exhibit sensitivity to thermal stimuli, which is often associated with pulpal inflammation. | Thermal sensitivity was introduced despite not being reported in the case | The model hallucinated a diagnostic symptom that was not present in the clinical data. |
| Bing | Suggested periodontal treatment despite findings consistent with pulpal necrosis and apical pathology. | Incorrect diagnosis-treatment match | The model produced a management plan inconsistent with the likely endodontic origin of the lesion. |
| Clova X | Suggested observation or symptomatic treatment despite findings consistent with pulpal necrosis. | Treatment-diagnosis mismatch | The proposed treatment strategy did not align with the likely endodontic pathology indicated by case information. |
