# Supplementary material for: Large Language Models for Endodontic Symptom Assessment and Treatment Planning Using Image-Free Clinical Records: Comparative Evaluation Study
Source: JMIR Med Inform. 2026 Jul 24;14:e86145. doi: 10.2196/86145 (PMC13399569; doi:10.2196/86145)
Supplement: Multimedia Appendix 7 [file medinform-v14-e86145-s007.docx]

| **Supplemental Table 7a.** Strict diagnostic metrics (sensitivity, specificity, PPV, NPV, and accuracy with 95% CI) of LLMs and human evaluators for pulpal disease, using only a score of 2 as a positive match | | | | | | | | |
| --- | --- | --- | --- | --- | --- | --- | --- | --- |
| N=58 | ChatGPT 4.0 | Gemini 1.5 Pro | Bing | Clova X | AGD Specialist | AGD Residents | Endodontic Residents | Senior Students |
| Sensitivity (95% CI) | 0.36 (0.24-0.50) | 0.28 (0.17-0.41) | 0.29 (0.18-0.43) | 0.26 (0.15-0.39) | 0.48 (0.41-0.56) | 0.37 (0.30-0.45) | 0.39  (0.31- 0.46) | 0.25 (0.19-0.32) |
| Specificity (95% CI) | 0.71 (0.55-0.84) | 0.55 (0.39-0.70) | 0.69 (0.53-0.82) | 0.57 (0.41-0.72) | 0.57 (0.48-0.66) | 0.65 (0.56-0.73) | 0.67  (0.58- 0.75) | 0.61 (0.52-0.70) |
| PPV^b^ (95% CI) | 0.63 (0.49-0.75) | 0.46 (0.33-0.59) | 0.57 (0.42-0.71) | 0.45 (0.32-0.59) | 0.61 (0.55-0.67) | 0.60 (0.52-0.67) | 0.60  (0.52- 0.64) | 0.47 (0.38-0.55) |
| NPV^c^ (95% CI) | 0.45 (0.38-0.52) | 0.35 (0.28-0.43) | 0.41 (0.35-0.48) | 0.36 (0.29-0.43) | 0.44 (0.39-0.50) | 0.43 (0.39-0.47) | 0.43  (0.39- 0.48) | 0.37 (0.33-0.41) |
| Accuracy (95% CI) | 0.51 (0.41-0.61) | 0.39 (0.29-0.49) | 0.46 (0.36-0.56) | 0.39 (0.29-0.49) | 0.52 (0.46-0.58) | 0.49 (0.43-0.55) | 0.50  (0.44- 0.55) | 0.40 (0.34-0.46) |
| ^a^CI, confidence interval; ^b^PPV, positive predictive value; ^c^NPV, negative predictive value; human evaluator metrics were calculated by combining responses across three evaluators per group (effective n=174 for pulpal, n=126 for periapical); large language model metrics reflect single-pass evaluations (n=58 for pulpal, n=42 for periapical). | | | | | | | | |

| **Supplemental Table 7b.** Strict diagnostic metrics (sensitivity, specificity, PPV, NPV, and accuracy with 95% CI) of LLMs and human evaluators for periapical disease, using only a score of 2 as a positive match | | | | | | | | |
| --- | --- | --- | --- | --- | --- | --- | --- | --- |
| N=42 | ChatGPT 4.0 | Gemini 1.5 Pro | Bing | Clova X | AGD Specialist | AGD Residents | Endodontic Residents | Senior Students |
| Sensitivity (95% CI) | 0.31 (0.18-0.47) | 0.12 (0.04-0.26) | 0.26 (0.14-0.42) | 0.12 (0.04-0.26) | 0.44 (0.36-0.54) | 0.29 (0.21-0.37) | 0.33  (0.25- 0.42) | 0.24 (0.17-0.32) |
| Specificity (95% CI) | 0.67 (0.54-0.79) | 0.62 (0.48-0.74) | 0.59 (0.45-0.71) | 0.72 (0.59-0.83) | 0.66 (0.59-0.73) | 0.64 (0.56-0.71) | 0.62  (0.54-  0.69) | 0.58 (0.50-0.65) |
| PPV^b^ (95% CI) | 0.41 (0.28-0.55) | 0.19 (0.09-0.36) | 0.31 (0.20-0.45) | 0.24 (0.11-0.44) | 0.49 (0.42-0.56) | 0.36 (0.29-0.45) | 0.39  (0.32- 0.47) | 0.29 (0.22-0.37) |
| NPV^c^ (95% CI) | 0.57 (0.51-0.64) | 0.49 (0.44-0.55) | 0.52 (0.45-0.59) | 0.53 (0.48-0.58) | 0.62 (0.58-0.66) | 0.55 (0.51-0.59) | 0.56  (0.52- 0.60) | 0.51 (0.47-0.55) |
| Accuracy (95% CI) | 0.52 (0.42-0.62) | 0.41 (0.31-0.51) | 0.45 (0.35-0.55) | 0.47 (0.37-0.57) | 0.57 (0.51-0.63) | 0.49 (0.43-0.55) | 0.50  (0.44- 0.56) | 0.44 (0.38-0.49) |
| ^a^CI, confidence interval; ^b^PPV, positive predictive value; ^c^NPV, negative predictive value; human evaluator metrics were calculated by combining responses across three evaluators per group (effective n=174 for pulpal, n=126 for periapical); large language model metrics reflect single-pass evaluations (n=58 for pulpal, n=42 for periapical). | | | | | | | | |
